# Challenges and Opportunities in Robotic Food Handling: A Review

**DOI:** 10.3389/frobt.2021.789107

**Published:** 2022-01-13

**Authors:** Zhongkui Wang, Shinichi Hirai, Sadao Kawamura

**Affiliations:** ^1^ Research Organization of Science and Technology, Ritsumeikan University, Kusatsu, Japan; ^2^ Department of Robotics, Ritsumeikan University, Kusatsu, Japan

**Keywords:** food property, robotic end-effector, food recognition, food database, food handling, automation

## Abstract

Despite developments in robotics and automation technologies, several challenges need to be addressed to fulfill the high demand for automating various manufacturing processes in the food industry. In our opinion, these challenges can be classified as: the development of robotic end-effectors to cope with large variations of food products with high practicality and low cost, recognition of food products and materials in 3D scenario, better understanding of fundamental information of food products including food categorization and physical properties from the viewpoint of robotic handling. In this review, we first introduce the challenges in robotic food handling and then highlight the advances in robotic end-effectors, food recognition, and fundamental information of food products related to robotic food handling. Finally, future research directions and opportunities are discussed based on an analysis of the challenges and state-of-the-art developments.

## 1 Introduction

The food industry is highly diverse and covers many industrial activities, such as production, processing, packaging, distribution, preparation, preservation, and food service. Traditionally, the food industry is considered to be low-tech, but it has become more technology intensive in recent years, as measured by its R&D to sales ratio (Traill and Meulenberg, 2002). Due to an aging society and labor shortages in countries such as Japan, automation in the food industry is in high demand to maintain profit margins. In particular, upon the impact of the COVID-19 pandemic, automation is strongly advocated at all stages in food production systems considering food safety and ensuring food supply (Henry, 2020).

In food factories, some operations such as food production, processing, and packaging require direct contact with food products. For operations of production and processing, dedicated machines, for example rice making machine, dumpling maker machine, and automatic chocolate molding machine, are usually preferable because the operations are constantly required without frequent change and update. In dedicated machines, handling operations of food materials or products can be specified and pick-and-place operations are often not required. On the other hand, packaging food products are generally conducted by pick-and-place operations. Moreover, pick-and-place operations are also required to transfer food products from one dedicated machine to another for connecting different processing operations. To realize pick-and-place operations, robotic systems consisting of robotic manipulator, end-effector, and sensors (*e.g.*, camera), are often used because of their efficiency and adaptability to various food products.

**FIGURE 1:**
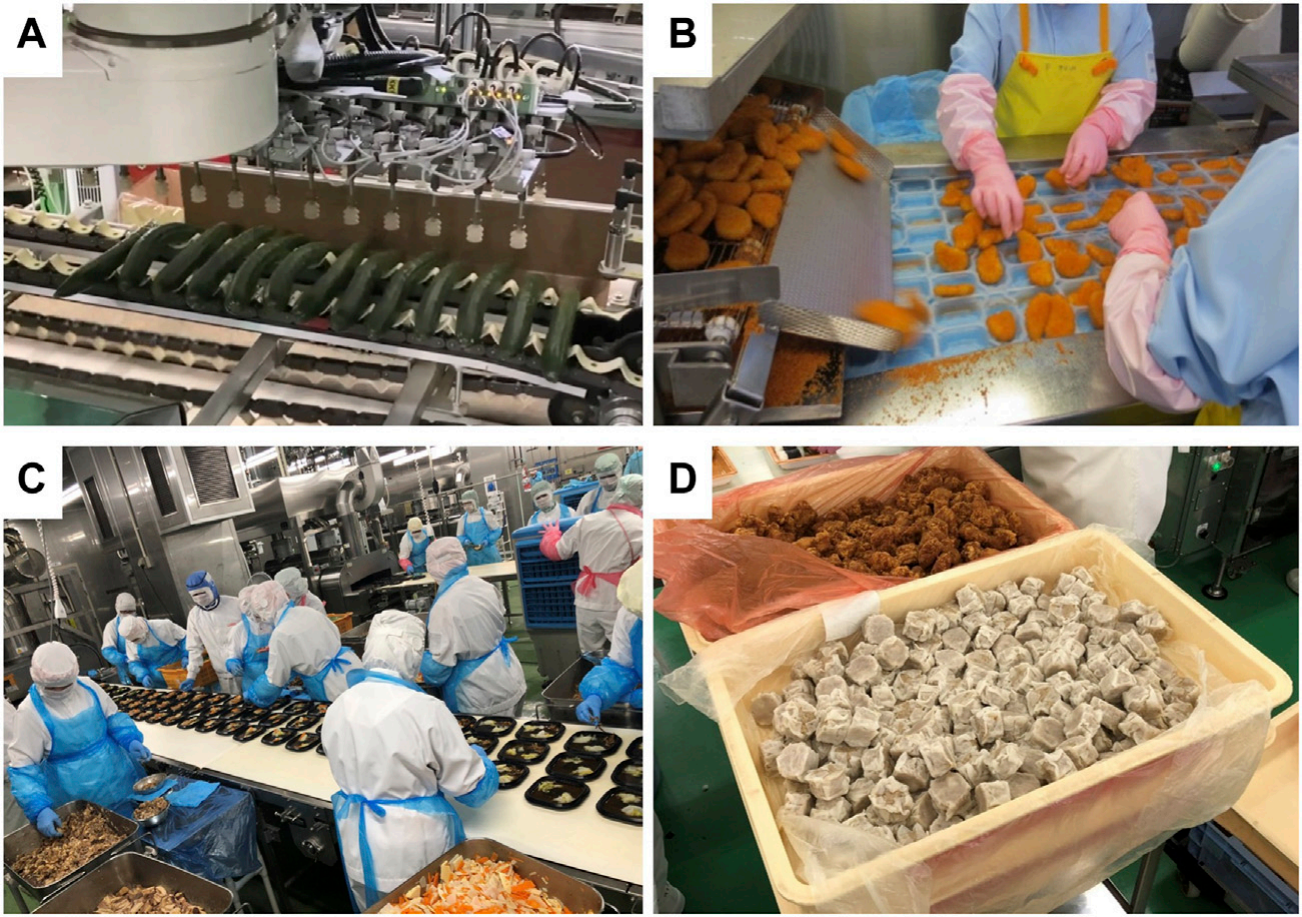
Factory scenarios of food handling operations in the food industry: **(A)** using suction cups to package cucumber, **(B)** human laborers packaging fried shrimps, **(C)** human laborers manufacturing Japanese boxed lunches, and **(D)** examples of prepared food materials in containers.

Currently, industrial robotic arms are used as manipulators in the food industry for generating desired motions and carrying payloads in pick-and-place operations (Bader and Rahimifard, 2020). The industrial robotic arms can provide high position accuracy and high motion speed, but often with high cost. For robotic end-effector, the most widely used ones in food factories are suction cups as shown in Figure 1A, which are inexpensive and can be operated simply. Simple operation is an essential requirement for food handling systems because most handling tasks must be completed within a short time period to maintain production efficiency. However, there are many food products or handling operations, such as grasping food materials with moisture and porous surfaces, cannot be performed using suction cups. As a result, these operations are eventually left for human laborers to perform, as examples shown in Figures 1B,C, and the automation of such operations is currently the main task for most enterprisers and researchers in the food industry.

To automate the pick-and-place operations as shown in Figures 1B,C, gripping-type robotic end-effectors are needed and they are required to adapt to the variations of food products considering the frequent changes and updates of food products. Moreover, in many scenarios, cooked and prepared food products are randomly distributed or stored in containers as shown in Figure 1D instead of aligning on a belt conveyor. This brings challenges for grasping, recognition, and sensing. To address these challenges, many researches have been carried out and numbers of commercialized systems are available. However, there are still many open issues to be challenged to further accelerate the automation in the food industry. Therefore, in this review, we attempt to address these challenges in details and review recent developments and advances regarding these challenges. Finally, we discuss potential opportunities and research directions for improving food handling automation in the food industry.

## 2 Challenges

There are many challenges in the development of automated systems to be used in the food industry. In this review, we focus on robotic systems to perform typical handling tasks, such as pick-and-place operations. It is a simple operation for human laborers, but it presents many difficulties for a robotic system to achieve an efficiency similar to that of human laborers. Many technologies are required to complete these simple operations, such as the technologies for successfully recognizing food items, effectively handling food products, and robotic end-effectors for handling various food products.

**FIGURE 2:**
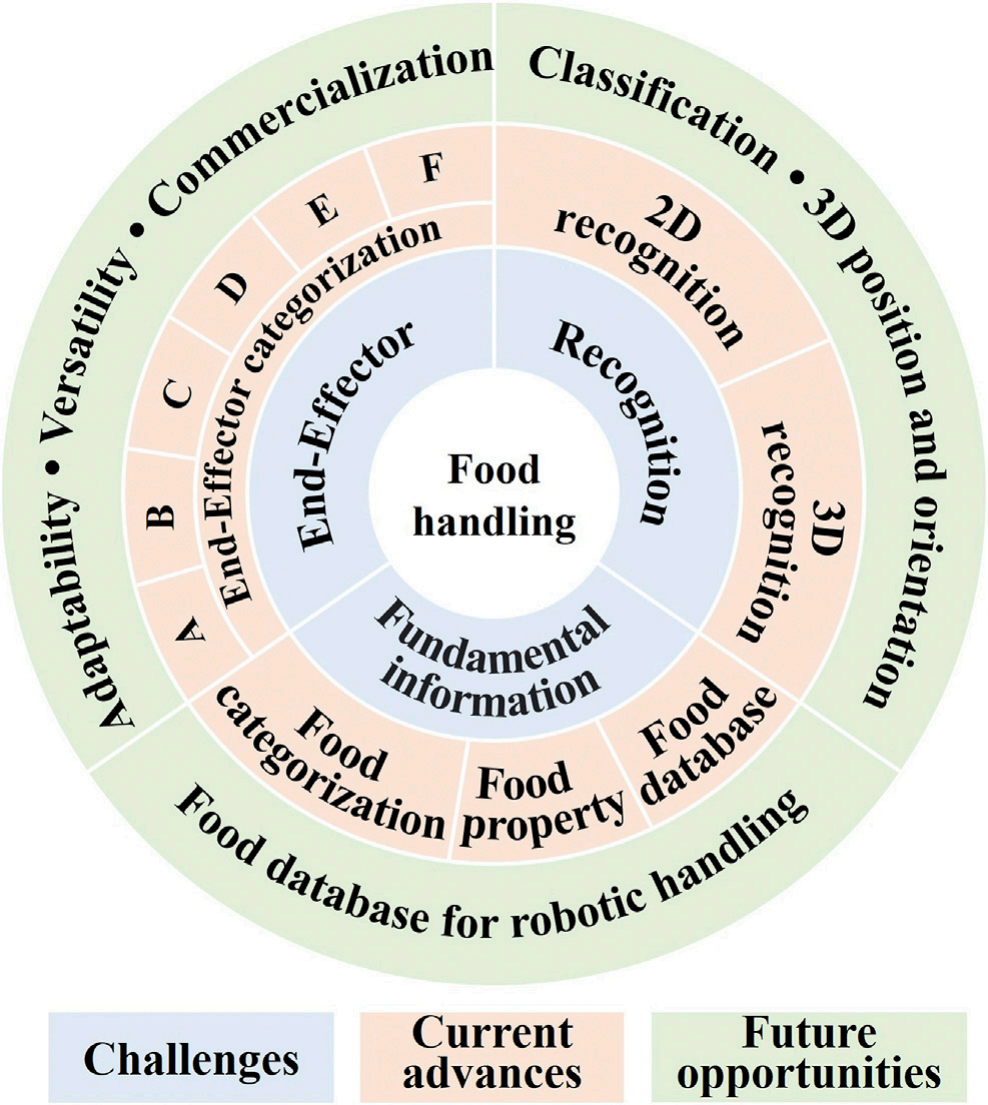
The structure of the review contents.

Food products are mostly non-rigid, sometimes fragile, and easily bruised and marked when they come in contact with hard surfaces. In addition, food products are susceptible to bacterial contamination, and their properties are highly affected by environmental conditions, such as temperature, humidity, and pressure (Chua et al., 2003). These characteristics of food products bring many challenges when developing robotic systems to handle them. In this review, we will focus on the following three aspects: robotic end-effector, food recognition, and fundamental information of food products, as indicated by the light blue area in Figure 2. More details will be discussed in the following sections.

### 2.1 Robotic End-Effectors

To cope with the large variety and variable characteristics of food products, various robotic end-effectors need to be developed. The lack of effective robotic end-effectors is considered one of the main reasons that hinder the rapid introduction of robots into the food industry (Chua et al., 2003). Lien (2013); Fantoni et al. (2014) summarized the challenges of developing robotic grippers for food handling tasks and suggested that robotic grippers must cope with the softness, uneven surfaces, and non-uniform shapes of food products, and fulfill the hygienic requirements. In addition, robotic end-effectors must also adapt to the food surface conditions, such as wet and sticky surfaces. In some scenarios where the spaces or gaps among food products are small, as shown in Figure 1B, robotic end-effectors must have the ability to enter the small spaces for grasping. From contamination considerations, the robotic end-effectors must contain as few mechanical components as possible to avoid dropping into the food product. From the system’s point of view, the robotic end-effectors must have a simple motion and can be operated at a high speed to achieve a proper takt time. Furthermore, the end-effectors should be low cost and designed to meet the hygienic design principles (EHEDG, 2018).

### 2.2 Food Recognition

Difficulties in food recognition vary significantly depending on the operation scenarios. In food factories, scenarios of food recognition can be divided roughly into two categories: 1) food products aligned or scattered on a food conveyor with no overlap, 2) food products randomly distributed in a food container with overlaps and contacts among food products, as shown in Figure 1B, which is also known as the random bin-picking (RBP) scenario. In the first scenario, food products or materials are separately located on a flat surface. Therefore, they can be recognized by using conventional 2D image processing methods based on color information or pattern-matching techniques. The position and posture of the food product need to be calculated only in a 2D plane. For such a scenario, automated robotic systems can often be found in food factories, for example, the robotic systems for picking pizza and packaging powder based on pattern matching (Connolly, 2007a). On the other hand, it is difficult to perform food recognition in the RBP scenario because the food products may overlap and are located in a 3D space in which the position and posture of the food product must be described. The 3D template matching technique can be used in 3D space to recognize the position and pose of objects with well-defined geometries (Vock et al., 2019), but it is difficult to be applied to food products that have large variations in geometrical parameters. Therefore, food recognition in the 3D or RBP scenario for various food products remains an challenging issue.

### 2.3 Fundamental Information

To achieve successful handling of food product, an effective handling strategy can be very helpful. For instance, the grasping force needs to be small enough to avoid damage on food product, but it must also be large enough to complete a pick-and-place task without dropping. Moreover, grasping velocity also plays an important role when considering the viscosity of food product and possible impacts upon grasping. To the best of the authors’ knowledge, there are very few researches focusing on investigating optimal handling strategies of food products. In actual applications, these handling strategies are usually pre-determined through trial-and-error experiments. The reason behind this is the lack of fundamental information to properly model the “engineering” properties of food products, such as size, shape, weight, softness, surface condition, friction coefficient, viscoelasticity, rheology, fragility, ease of bruising, and so on. Researches in this area, especially from the viewpoint of robotic handling, have not been carried out frequently. There are specific machines or devices used for measuring these properties for various research purposes, such as food science, nutrition, and mastication. Unfortunately, such data for the purpose of robotic handling are barely available, but they are essentially important for designing end-effectors and investigating grasping strategies. In addition, handling strategy depends on robotic end-effector and food target. To improve versatility of handling strategy, categorizations of robotic end-effectors and food products based on their characterizations are also essential, and such research activities have not been carried out frequently so far.

## 3 Recent Advances

To tackle the above-mentioned challenges, many researches and commercial robotic systems have been developed in the last few decades. In this section, we review these advances as indicated with the light orange area in Figure 2.

### 3.1 Advances in Robotic End-Effectors

**FIGURE 3:**
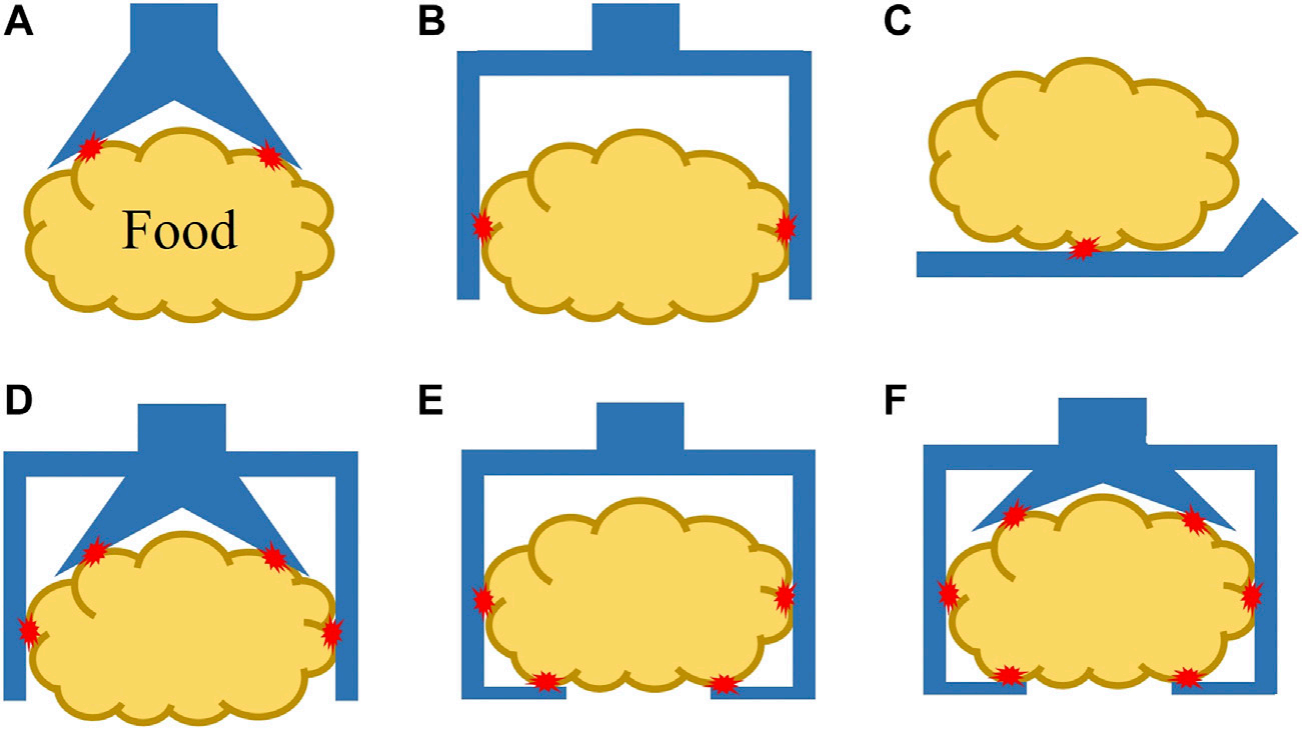
Different types of robotic end-effectors according to their handling positions at **(A)** top surface, **(B)** side surface, **(C)** bottom surface, **(D)** top and side surfaces, **(E)** side and bottom surfaces, and **(F)** top, side, and bottom surfaces. Red star marks indicate contact positions.

Many robotic hands and grippers have been proposed and studied so far to handle food products and materials. To better review and address related work, we divided robotic end-effectors into six categories, as shown in Figure 3, based on the position/positions where an end-effector contacts the target food product. We employ this classification approach instead of using grasping principles as nicely reviewed in (Fantoni et al., 2014) because we tend to focus on the forces that the food target may be received and reduce the number of categories.

#### 3.1.1 Grasping at Top Surface

In the case of grasping food products from the top surface (Figure 3A), conventional grippers can be roughly categorized into four groups: 1) suction cups, 2) grippers using the Bernoulli principle (Petterson et al., 2010) or the Coanda effect (Elango et al., 2012, 2018), 3) devices using adhesion force from a roller (Davis et al., 2007) or by freezing the moist surface (Lien and Gjerstad, 2008), and 4) gripper penetrating inside the food product by needles (Gjerstad et al., 2006). A suction cup with vacuum pressure has the advantages of a simple structure and stable grasp. However, suction cups usually require the surface of the food product to be relatively flat, smooth, and dry. In addition, it may leave a bruise or mark on the product surface when handling raw food products. To overcome these disadvantages, grippers based on the Bernoulli principle or Coanda effect have been proposed, and these grippers do not require direct contact with food products for grasping. However, the grasping becomes unstable, and rotation motion may be generated owing the high-speed air flow. Grippers using adhesion force are usually used for specific food products with sticky properties (*e.g.,* pasta dough) for the roller type and moist surfaces (*e.g.,* fresh fish piece) for the freezer type. The freezer-type gripper also requires a sharp knife mechanism or heat flow to release the food product upon placement (Fantoni et al., 2014).

In recent years, new types of grippers have also been proposed for grasping various objects, including food products. Amend et al. (2016) proposed jamming grippers and successfully tested them for grasping various types of objects. They appeared to be very promising for practical applications in the food industry. Unfortunately, commercialization failed due to challenges such as leaks, difficult actuation and assembly, and materials. Koivikko et al. (2021) developed soft suction grippers with switchable stiffness to achieve both small and large forces. The grippers were tested on fruits, such as mangoes and bananas. Recently, a pneumatically driven needle gripper was also proposed to grasp raw food materials from their top surface (Wang et al., 2021c). However, these grippers are still in their research stages, and commercialization and applications in the food industry are expected only in the future.

#### 3.1.2 Grasping at Side Surfaces

Grippers belonging to this group have been widely studied and are frequently applied in the food industry. When grasping an object at its side surfaces, the object size must be known, and the gripper must provide sufficient stroke for successful grasping. Because the friction force dominates the grasping performance, it is important to ensure a sufficient friction force for stable grasping. This type of grasping has the advantage of better placement accuracy because the object is enclosed inside the gripper, and the posture of the object can be easily adjusted. Many conventional two-fingered or multi-fingered parallel grippers for manufacturing automation have been modified for application in the food industry after solving the food compatibility issues and ensuring that there is no damage to the food products. One good example is the SCHUNK food gripper (SCHUNK, 2021), which provides customized, fully regulation-compliant components and gripper solutions for the food industry. In recent years, along with the rise of soft robotics, many soft robotic grippers have been developed for handling food products. Soft grippers have the advantages of easy adaptation to food variations and because of their soft bodies less risk of damage to the food products. Examples of commercialized soft grippers include the mGrip grippers from Soft Robotics, Inc. (Robotics Inc., Soft, 2021), soft gripper from OnRobot (OnRobot, A/S, 2021), a modular-designed soft gripper from SoftGripping (SoftGripping, 2021), and the soft flexible gripper from Soft Robot Technology Co. Ltd. (SRT., 2021). These grippers are pneumatically driven and fabricated with silicone materials. They are able to handle a wide range of irregular shaped and delicate food products. In addition, Festo (Festo, 2021a) provides a shape adaptive gripper using soft Fin Ray^®^ structure which can passively adapt to the shape of a target object.

In addition to these commercialized end-effectors, many studies have been conducted to design end-effectors for handling challenging food products. Pettersson et al. (2010) developed a soft gripper using the effects of magnetorheological fluid to cope with the ease of bruising and shape variations of food products, such as apples, carrots, strawberries, broccoli, and grapes. Maruyama et al. (2013) proposed a robotic gripper made from an incompressible fluid enclosed in a rubber part to grasp fragile objects, such as potato chips and tofu. Endo and Otomo developed a two-degree-of-freedom multi-fingered gripper for grasping noodles and simmered food by considering an appetizing presentation (Endo and Otomo, 2016). To handle similar chopped and granular food materials, Kuriyama et al. (2019) proposed a pneumatically driven wrapping gripper for automating the topping operation of a Japanese lunch box. Blanes et al. (2014) proposed several pneumatic actuators and mechanisms fabricated by additive manufacturing for food handling. These grippers have promising capabilities for handling specific food materials, but many requirements, such as food compatibility and hygiene, need to be considered before they can be commercialized or used in actual applications.

#### 3.1.3 Grasping From Bottom Surface

Handling food products solely from the bottom surface can often be seen in kitchens when using a spatula to cook foods. However, this approach is not widely adopted in automation systems because of the possible instability or slippage during high-speed translation motion. The only commercialized robotic end-effector using this principle is the SWITL hand developed by FURUKAWA KIKO (Furukawa, 2021). The hand consisted of a Teflon film and a stainless-steel plate. The Teflon rotates around the plate like a belt conveyer and creeps under the food material. It can handle various food materials, such as slices of ham and cheese, semi-liquid-gels, mousses, and mayonnaise sauce or melted cheese (Kusuda, 2011).

#### 3.1.4 Grasping at Top and Side Surfaces

It is natural to combine different principles to improve the grasping capabilities. In this subsection, we review related work on end-effectors that combine the grasping at both the top and side surfaces of an object. However, we do not consider those that combine both principles, but they are operated independently by using a tool changer. In this end-effector group, it is often possible to observe the combination of suction and gripping. A commercialized example is the gripper from RightHand Robotics, Inc., which consists of a suction cup and a three-fingered gripper (RightHand Robotics, 2021). Another commercialized example is the TentacleGripper from Festo, which is structured as a silicone tentacle and two rows of suction cups located on the surface of the tentacle mimicking the octopus leg (Xie et al., 2020; Festo., 2021b). In addition, researchers have proposed other types of grippers that combine suction and gripping principles. Bryan et al. (2019) proposed a soft robotic gripper with three cable-driven fingers, and each finger is equipped with three suction cups. Multi-fingered grippers with suction cups at the fingertips have also been developed by researchers for handling various objects, including food materials (Yamaguchi et al., 2013; Wang et al., 2020). Moreover, soft grippers with enveloping motion have also been proposed to grasp various objects, including fragile food products (Li et al., 2019; Hao et al., 2021). The gripper, upon grasping can form an enclosed space; therefore, the gripper itself works as a suction cup when vacuum pressure is applied (Hao et al., 2021). Both grippers can adapt to the object shape and generate large grasping forces. In addition, grippers integrated with gecko-inspired adhesives have been proposed by researchers to improve grasping performance (Song et al., 2014; Glick et al., 2018). These grippers combine the principles of gripping and adhesion, and require the contact surface to be relatively dry to improve the adhesion performance. Some of the above-mentioned grippers may not be designed specifically for food industry applications, but the approaches used to achieve stable grasping can be extended to handle food products.

#### 3.1.5 Grasping by Side and Bottom Surfaces

There are also robotic end-effectors that combine grasping from the side and bottom surfaces. Ma et al. (2020) proposed a flat-shaped paper gripper for food grasping. This gripper can slide a thin paper sheet under the food product for grasping, and the puller sheet can also make contact with the food product for stabilization purposes. Gafer et al. (2020) developed a quad-spatula gripper for handling food ingredients. This gripper has four cable-driven fingers with a spatula-shaped plate at each fingertip. When grasping, the plates scoop the food ingredients, and the fingers also apply a grasping force. In addition, Wang et al. (2021a) proposed a scooping-binding gripper to handle various food products, especially those with a low height profile and slippery properties. The gripper consists of two thin scooping plates and multiple rubber strings. Upon grasping, the scooping plates are inserted under the bottom surface of the food product and the rubber strings wrap around the side surfaces to stabilize the grasp. However, no commercially available end-effectors were found that use this combination of grasping by the side and bottom surfaces of food products.

#### 3.1.6 Grasping by Top, Side and Bottom Surfaces

The last group of grippers achieves stable grasping by enveloping food products from all surfaces. One commercially available gripper of this type is the meat gripper (AppliedRobotics., 2021). This gripper consists of two L-shaped grip plates to grasp food products and a center plate at the top with a passive spring mechanism to provide a pushing force for stabilization. The gripper is manufactured from lightweight materials approved by the FDA and USDA, and it can be used to handle various food products, such as fresh meat, fish, cheese, bacon, and many other nonuniform products. For research activities, Sam and Nefti proposed a multifunctional gripper for handling various food products (Sam and Nefti, 2010). This gripper was constructed using a suction mechanism and four rigid fingers. The suction mechanism was based on the Bernoulli effect. After the food product was held by the suction mechanism, the four fingers were closed to envelop the food product for stabilization.

### 3.2 Advances in Food Recognition

#### 3.2.1 2D Recognition

For the scenario of food scattered or aligned on food conveyor, recognition can be performed using 2D image processing or pattern matching approaches, which have been integreated in commercially available robotic systems, such as the ABB FlexPicker Packaing Robot (ABB, 2021). In addition, 2D image processing can be also used in recognizing certain food products with overlapping and occluding (Muhammedali et al., 2004). The approach of 2D image processing assumes the depth information of the food target is known and only the position and orientation in the horizontal plane (usually the food conveyor) need to be recognized. Using only image processing can greatly reduce the complexity of the recognition problem and results in an efficient solution. However, it is difficult to be applied to recognize multiple food products or materials of different categories. For recognizing multiple targets, machine learning based approaches are often adopted. Kawano and Yanai developed a smartphone-based food recognition system (FoodCam) to estimate calories and nutrients in foods and record a user’s eating habits (Kawano and Yanai, 2013; Kawano and Yanai, 2014b; Kawano and Yanai, 2015). It achieved a classification rate of 79.2% for the top five category candidates for a 100-category food dataset (Kawano and Yanai, 2015). Liu et al. (2018) proposed a deep-learning-based food recognition system for dietary assessment on an edge computing service infrastructure and achieved approximately a 90% classification accuracy with three different food datasets. In addition, Ciocca et al. (2014) developed a food recognition system that can track the eaten food and the user’s dietary habits, realize automatic billing procedure based on the recognized foods, and evaluate the leftovers for better estimation of food intake. Even though these approaches are not proposed for robotic handling purposes, the ideas of food segmentation and classification can be extended to applications of robotic handling.

#### 3.2.2 3D Recognition

3D recognition is commonly required in the RBP scenario, as an example shown in Figure 1B. The RBP problem has been widely studied for grasping rigid parts with known CAD models in industrial applications (Liu et al., 2012). Many commercial systems are available to solve the RBP problem. Many robot manufacturers provide such vision systems together with their robotic manipulators, such as the FANUC 3D Vision Sensor (Connolly, 2007b; FANUC., 2021), MELFA-3D Vision from Mitsubishi Electric (MITSUBISHI ELECTRIC, 2021), and KUKA.PerceptionTech (KUKA., 2021). There are also companies that provide specific 3D vision systems for RBP tasks, such as the 3D vision system from Pickit (Pickit., 2021) and the 3D vision sensor “TVS” from Kyoto Robotics (KYOTO ROBOTICS, 2021). However, most of these approaches assume dealing with objects of known and non-deformable shapes (Castaman et al., 2020), and therefore, they have not yet been applied to food products. Machine learning-based approaches have been investigated to recognize food products in the RBP scenario. Joffe et al. (2019) proposed a method using a standard Faster R-CNN architecture with a Resnet 101 feature extractor to recognize chicken and evaluated two pose estimation approaches: the augmented autoencoder and direct regression approach. A suction cup gripper was used to pick the chickens. Nishina and Hasegawa proposed an approach to obtain the optimal grasping points through a deep neural network and successfully applied it to a two-fingered robotic hand and a suction cup gripper (Nishina and Hasegawa, 2020). Moreover, Low et al. (2021) proposed a YOLOv3 based object detection algorithm for recognizing sousage, potato, broccoli, and tomato in a RBP scenario. The accuracy of object classification and the speed of pose estimation were achieve at 67.06% and 92.7 ms, respectively.

### 3.3 Advances in Fundamental Information

#### 3.3.1 Food Categorization

Food products have large varieties and big variations in shape, size, weight, surface conditions, softness, and other physical properties. To maximize the cost performance of a robotic end-effector, it is essential to investigate how many categories of food products the end-effector can handle. Unfortunately, it is impractical to perform experimental tests on each food product considering the large varieties. Therefore, food categorization needs to be carried out from a viewpoint of robotic handling. Erzincanli and Sharp (1997) proposed a classification system for robotic food handling and categorized food products depending on their shapes, dimensions, surfaces, compiance, temperature, and weight. The food shape was classified into eight groups with standard geometries, such as flat, cylinder, square, ellipse, and so on. The surface and compliance were qualitatively described as smooth, furry, thorny, rigid, semi-rigid, non-rigid, and so on. In addition, Wurdemann et al. (2011) presented a categorization system for classifying food products to assist food ordering process. Key characteristics used for the classification are symmetry, surface condition, hardness, springiness, and resistance to damage. All these characteristics are also defined as qualitative descriptions.

#### 3.3.2 Food Properties

Food properties have been studied for many decades. Among the different properties, elasticity, often indicated by Young’s modulus, has been widely studied for various purposes. Williams et al. (2005) experimentally studied the Young’s modulus of a series of foods, ranging from apple pulp to prune pit, to develop a primate masticatory apparatus. Ogawa et al. (2015) investigated the changes in Young’s modulus and Poisson’s ratio in Japanese radish and carrot roots during boiling to assess food quality. Kadowaki et al. (2013) measured the Young’s modulus of crispy foods at the microscopic level to study food crash. Recently, Sinha and Bhargav studied the effects of experimental parameters, such as the deformation rate, sample shape and size, and moisture content, when measuring the Young’s modulus of potato and sweet potato samples for food quality assessment (Sinha and Bhargav, 2020). In addition to elasticity, viscoelastic or rheological properties were also investigated, and different analytical models were proposed to capture the complex deformation and force behaviors. The linear viscoelasticity of gummy candy, Mozzarella cheese, and cooked ham was characterized by Singh et al. (2006) using broadband viscoelastic spectroscopy (BVS). Sakamoto et al. (2007, 2009) studied the viscoelasticity of Japanese food “Norimaki” using Maxwell and Burger models to realize the optimum design of robotic handling. Rheological models were also investigated by Wang and Hirai (2010, 2012) to simultaneously capture both deformation (especially, residual deformation) and force of Japanese sweets.

Friction, as a surface property, has mostly been investigated for studying the oral mastication sensation. For example, Joyner et al. (2014) proposed a double-ball tribological system to evaluate the friction of acid milk gels with and without the addition of saliva. Chojnicka-Paszun and de Jongh explored the tribological properties of food-relevant aqueous solutions on different surfaces to study the mastication of food products (Chojnicka-Paszun and de Jongh, 2014). A universal mechanical tester “Tribolab” was used to measure the friction force between the polydimethylsiloxane (PDMS) surface, which mimics the oral surface, and intact soft solid foods such as gelatin gels and sausages (Fuhrmann et al., 2020). In addition, a measuring apparatus mimicking a robotic grasping scenario was developed to measure the friction coefficient between a flat stainless plate and food material (Wang et al., 2021b).

Food geometry is another important data for assisting with robotic handling. In particular, geometry is essential to study robotic grasping in a simulation environment or when performing 3D template matching for food recognition. Balcerzak et al. (2015) proposed an approach to construct a geometric model of agri-food products using the Autodesk 3ds Max to predict the behavior of agri-food products subjected to drying, cooling, and heating operations. Goñi et al. (2008) investigated three-dimensional geometric models of lamb, pork, and chicken carcasses through magnetic resonance imaging for food process modeling applications. Research related to food quality inspection also employs the shape information of food materials. Weres et al. (2009) developed software packages to assess quality and classify selected agri-food products, to represent their 3D geometry, and visualize their property changes during thermo-mechanical processing. Ding and Gunasekaran developed an automated food shape inspection system that included a feature extraction stage and a classification stage, and tested it on corn kernels, almonds, and animal-shaped crackers (Ding and Gunasekaran, 1994). In addition, Loebnitz and Grunert conducted a survey to explore the effect of food shape abnormality on purchase intention and how environmental concern and social trust might moderate this intention (Loebnitz and Grunert, 2015).

#### 3.3.3 Food Database

In terms of the food database, we often found those related to nutrient profile (U.S. Department of Agriculture, 2021), food composition (Food Standards Australia and New Zealand, 2021), and food constituents, chemistry, and biology (The Metabolomics Innovation Centre, 2021). Unfortunately, there are no databases directly applicable to robotic handling tasks. Available food databases are mainly developed for recognizing food products from 2D images and conducting calculations of calorie or nutrition for health monitoring purposes. Kawano and Yanai created a series of datasets for Japanese food (Kawano and Yanai, 2021). They first released a dataset (UEC FOOD-100) containing 100 types of food photos with a bounding box for each photo to indicate the location of the food item (Matsuda et al., 2012). The dataset was then extended to UEC FOOD-256 which contains 256 types of food photos (Kawano and Yanai, 2014a). Recently, the authors updated the UEC FOOD-100 dataset to the UEC-FoodPix Complete dataset with manually refined segmentation masks to enable accurate food segmentation (Okamoto and Yanai, 2021). In addition, the authors created a school lunch dataset containing 3940 multiple-dish images with bounding boxes on 21-class labels (Ege and Yanai, 2017). Furthermore, there are also datasets of Food-101 (Bossard et al., 2014) and Google Food-201 (Myers et al., 2015) for the recognition of Western food items, and a dataset for Chinese food identification (Chen et al., 2012). Even though these databases are not created directly for the purpose of robotic handling tasks, they have the potentials to be used for classifying food categories.

## 4 Future Opportunities

With an aging society and resulting labor shortages, advancements in robotic technologies will lead to the introduction of an increasing number of robotic systems into the food industry to replace human laborers performing simple tasks. There are plenty of opportunities (green area in Figure 2) for researchers and enterprises in the fields of robotic manipulators, robotic end-effectors, computer science, artificial intelligence, and system integration. In this study, we did not review robotic manipulators because industrial robots are commonly used in the food industry (Bader and Rahimifard, 2020), and research on new robotic manipulators specifically for the food industry is scarce. However, this does not mean that there is no need to develop novel robotic manipulators for industrial food applications. In fact, the majority of industrial robotic manipulators are not well suited to the specific needs of industrial food applications (Masey et al., 2010). In particular, the high cost of the current robotic systems presents a financial obstacle for food manufacturers. As summarized by Masey et al. (2010), robotic manipulators designed for the food industry should fulfill the requirements of easy to clean hygienic design, low cost, fast operational pick-and-place speed, safe operation alongside human workers, and easy to reprogram. In recent years, collaborative robots have been employed frequently in various applications, and they can meet the requirements of operating alongside human workers and are easy to program. However, the requirements of low cost and fast operational speed remain to be fulfilled.

Regarding to robotic end-effectors, research has been carried out intensively in recent years, along with the rise of the soft robotics field. However, there are still few commercialized end-effectors, and successful user cases in the food industry are even more limited. To be successful, the end-effectors must not only meet all the hygienic design principles and takt time requirements, but also be low cost and easy to be integrated into the existing robotic systems. Moreover, one end-effector should be able to handle various food products to adapt to rapid changes or updates of the target products in an automation line. Theoretical and experimental investigations need to be carried out to establish relationships between robotic end-effectors and food products. In addition, considering the grasping principle, as summarized in Table 1, only end-effectors grasping at the top and side surfaces are well studied and commercialized, but end-effectors grasping from the bottom surface and all surfaces have been barely studied so far. However, such end-effectors are required for handling various kinds of food products, especially slippery, heavy, and low-profile food products. An increasing number of such end-effectors are expected.

**TABLE 1:** Summarization of robotic end-effectors for food handling.

Gripper type	Commercialized	Under research
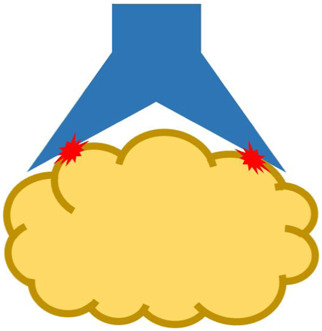	• Suction cup	• Adhesion using roller Davis et al. (2007)
	• Suction gripper based on Bernoulli’s principle VUOTOTECNICA. (2021)	• Freezing moisture surface Lien and Gjerstad. (2008)
	• Jamming gripper Amend et al. (2016)	• Needle gripper Gjerstad et al. (2006); Wang et al. (2021c)
		• Suction gripper with switchable stiffness Koivikko et al. (2021)
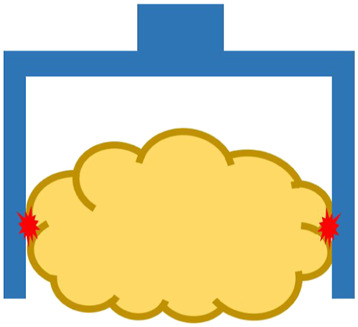	• SCHUNK food gripper SCHUNK. (2021)	• Gripper using magnetorheological fluid Pettersson et al. (2010)
	• mGrip gripper Robotics Inc., Soft. (2021)	• Gripper using incompressible fluid Maruyama et al. (2013)
	• OnRobot gripper OnRobot, A/S. (2021)	• Multi-fingered gripper Endo and Otomo. (2016)
	• Shape-adaptive gripper Festo. (2021a)	• Wrapping gripper Kuriyama et al. (2019)
	• Modular-designed gripper SoftGripping. (2021)	• 3D printed gripper Blanes et al. (2014)
	• Soft flexible gripper SRT. (2021)	
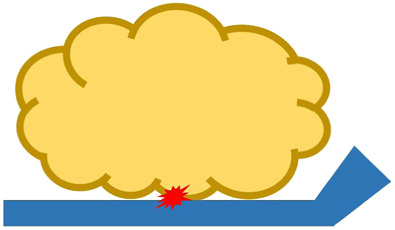	• SWITL hand FURUKAWA. (2021)	
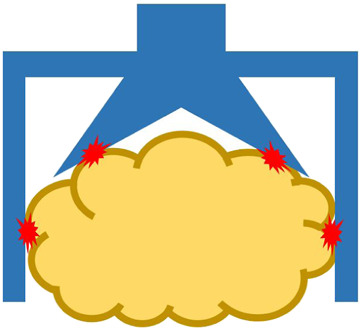	• RightHand gripper RightHand Robotics (2021)	• Cable-driven gripper with suction cups Bryan et al. (2019)
	• TentacleGripper Festo. (2021b)	• Pneumatic gripper with suction cups Wang et al. (2020)
		• Enveloping gripper Li et al. (2019); Hao et al. (2021)
		• Gecko-inspired gripper Song et al. (2014); Glick et al. (2018)
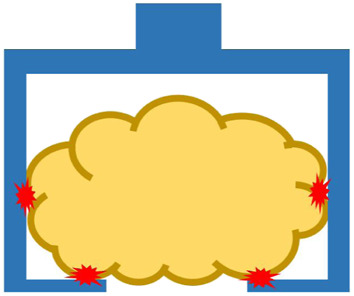		• Flat-shaped paper gripper Ma et al. (2020)
		• Quad-spatula gripper Gafer et al. (2020)
		• Scooping-binding gripper Wang et al. (2021a)
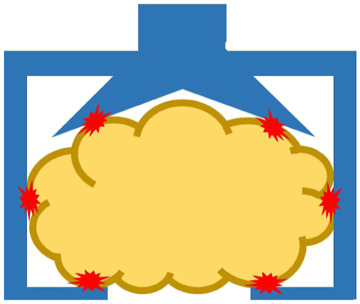	• Meat gripper AppliedRobotics. (2021)	• Multi-functional gripper Sam and Nefti. (2010)

For food recognition, the RBP scenario should be further investigated for various food products and materials. Attention need to be paid not only for classification and recognition, but also for the determination of the grasping position and orientation. In the bin-picking scenario, these positions and orientations are in three-dimensional space, and the accuracy of the recognition and determination may significantly affect the grasping performance. To improve the recognition accuracy, one potential approach is to use deep learning methods that learn representational features from the dataset during the training process and demonstrate stronger ability than traditional methods (Zhou et al., 2019). The databases mentioned in Section 3.3.3 may be used for developing deep learning models, but new databases for bin-picking scenarios are also needed to ensure satisfactory performance.

Regarding food categorization, it would be better to have a categorization system including quantitative descriptions of features such as surface condition, hardness, and resistance to damage. Relationships between robotic end-effectors and food categories need to be established to help the selection of proper robotic end-effector. For the physical properties and database of food products, it would be helpful to have a database containing food property information related to robotic handling, such as the properties of viscoelasticity or rheology, friction, geometry, and weight, similar to the food version of the Yale-CMU-Berkeley dataset (Calli et al., 2015, 2017). The properties should be presented in a way that facilitates robotic handling tasks. Such a database can lay a foundation for the development of robotic systems for food handling and can also be used to study and analyze robotic systems in a simulation environment. It may also inspire researchers to develop property-measuring devices and establish evaluation standards for various robotic end-effectors.

Finally, from a system’s point of view, a compact system with portability is preferable to cope with the high-mix low-volume production in the food industry. In addition, system engineers are usually not available in food factories; therefore, the robotic system used in such factories must have an easy-to-use interface, and complex system maintenance should be avoided or done automatically. Along with the development of the Fourth Industrial Revolution (4IR or Industry 4.0), an increasing number of industrial and home devices are connected through the technologies of the Internet of Things (IoT) or cyber physical systems (CPS) (Khan et al., 2018). Therefore, it is also a good opportunity to apply such technologies to the robotic systems in the food industry to extend their capabilities of machine-to-machine communication, self-monitoring, and automatic system updating.

## 5 Conclusion

The food industry has a very long history, but it is still a labor-intensive industry. There are many benefits to fully automate food preparation and processing operations, but difficulties are deep-rooted, and new technologies need to be integrated together to make a step forward. In this review, we investigated a large number of research articles and commercial systems related to the robotic handling of food products. We first summarized the basic challenges faced in the food industry for introducing robotic systems, and then elaborated on the advances in different aspects, such as the robotic end-effector, food recognition, and fundamental information of food categorization, property, and database, which are essentially important for developing robotic systems. Finally, we suggest future directions that are potentially promising to tackle these challenges and eventually help the process of automation in the food industry. The purpose of this review is to encourage researchers and enterprises in this field to further advance the existing technologies, develop new technologies, and put them into practice for automating various operations in the food industry.
